# Editorial: Chemical reactions and catalysis for a sustainable future

**DOI:** 10.3389/fchem.2023.1228591

**Published:** 2023-06-02

**Authors:** José C. S. dos Santos, Namasivayam Dhenadhayalan, Yanwei Li, Jose Luis Pinilla

**Affiliations:** ^1^ Instituto de Engenharias e Desenvolvimento Sustentável, Universidade da Integração Internacional da Lusofonia Afro-Brasileira, Redenção, CE, Brazil; ^2^ Department of Chemistry, National Taiwan University, Taipei, Taiwan; ^3^ Environment Research Institute, Shandong University, Qingdao, China; ^4^ Instituto de Carboquímica-CSIC, Zaragoza, Spain

**Keywords:** chemical reactions, catalysis, sustainable future, Research Topic, editorial

Developing catalytic chemical processes for a sustainable future is a constant challenge involving different knowledge áreas (Sakakura et al., 2007; Yang et al., 2013; Götz et al., 2016; Tian et al., 2023; Yu et al., 2023). It requires multidisciplinary actions that include economic sectors, industry, society, and the environment (Lee et al., 2006; Corma et al., 2007; Naik et al., 2010; Ferreira Mota et al., 2022; Tafete and Habtu, 2023). Furthermore, catalytic chemical processes require constant evaluation to achieve greater sustainability, mainly when applied in industries or on a domestic scale (Chheda et al., 2007; Khodakov et al., 2007; Oh et al., 2016; Deng et al., 2023). Indeed, chemical catalysis is inherent in developing a sustainable future (Kondratenko et al., 2013; Deng et al., 2023). Chemical reactions are inseparable from our subject since they are applied in different processes, such as the preparation of fuels, food, drugs, and energy (Arcadi, 2008; Lima et al., 2022; Moreira et al., 2022; Sales et al., 2022; de Sousa et al., 2023; Faizan and Song, 2023; Nogueira et al., 2023).

In this context, we include scientific research to help make these processes more sustainable. Moreover, consequently, it reduce the negative impact on the environment (Wang et al., 2023a; Zhu et al., 2023). Regarding chemical catalysis, reducing the amount of energy involved in the processes is fundamental (Roy et al., 2010; Yang et al., 2023a; Nogueira et al., 2023). This has a positive impact on reducing the use of polluting energy sources so that these systems can happen, such as the use of petroleum-derived fuels (Torborg and Beller, 2009; Catumba et al., 2023; Jafarian et al., 2023; Park and Kim, 2023). Furthermore, the greater need to use high temperature and pressure conditions increases energy consumption and, consequently, the production of waste that pollutes the environment (Singh et al., 2018; Djandja et al., 2023). Thus, chemical catalysis must seek to reduce the energy required for these processes, for example, in the design of robust catalysts (Yang et al., 2023b); The design of robust catalysts for industrial applications can be presented in different physical states, such as solid, liquid, or gaseous (Mariscal et al., 2016; Ferreira Mota et al., 2022; Issaka et al., 2023). The principle of sustainable functioning of these catalysts must include the formation of desired products (Centi et al., 2013). Be highly efficient in guiding molecules of reagents to the formation of desired products and eliminating the generation of unwanted waste (Li et al., 2023a; Li et al., 2023b). Another crucial factor for the design of sustainable catalysts must include their stabilization power, that is, whether the catalyst can be used repeatedly in the same reused process, minimizing the formation of polluting species and being economically viable (Centi et al., 2013; Wang et al., 2023b). In this way, the formation of sustainable reaction processes is the realization of catalytic systems on a large scale (Abbas-Abadi et al., 2023; Abuzeyad et al., 2023). This strategy makes it possible to reduce energy and polluting waste generated in the environment (Zhao et al., 2023). This fact also implies a decrease in energy use from oil and contributes to green chemistry practices (Goyal et al., 2008; Akram et al., 2023).

Therefore, catalysis is different in advancing clean, renewable, and consequently sustainable technologies (Waseem et al., 2023; Zhang et al., 2023). In this context, catalysis is fundamental in producing fuel cells, which convert chemical energy into electrical energy in an environmentally sustainable way (Zhao et al., 2015; Gong et al., 2023). Likewise, catalysis is fundamental in producing biofuels from renewable sources such as biomass (Li et al., 2023c; Jiang et al., 2023; Yu et al., 2023).

Scientific research to design catalytic, environmentally sustainable, and efficient chemical processes advances today. In this context, the work by Wang et al. (2023a) (Wang et al.) developed prepared WxCeMnOδ/3DOM ZrTiO_4_ catalysts with application possibilities for the simultaneous elimination of soot particulate matter and oxides of nitrogen from diesel engine exhaust, considering its characteristics such as ease of preparation, reduced costs, and high catalytic activity (Wang et al.). The prepared materials were analyzed, and the results showed high catalytic and structural activity. This high catalytic power is justified by its perfect structure, abundant acid sites, large surface area, and the synergistic effect between the active components. The prepared catalyst, W1CeMnOδ/3DOM ZrTiO_4_ exhibited overall thermal stability (250°C–396°C) at the lowest temperature for 90% NO conversion but also had the highest NO conversion rate (52%) at the combustion temperature of soot (Wang et al.).

The reduction of greenhouse gas emissions is a constant concern. This fact contributes to developing efficient catalytic processors that collaborate to reduce these effects. In this context, the work by Santiago et al. presented a proposal to convert CO2 into valuable chemicals, such as methanol (MeOH) and dimethyl ether (DME), by the medium of catalytic hydrogenation in catalysts based on Cu, Zn, and Al (Santiago et al.). In this approach, the researchers demonstrated insights into the reaction mechanism provided by the CO_2_ and H_2_ adsorption isotherms on the catalysts. The catalytic activity, conversion, and yields studied were correlated with the adsorption capacity of the reagents, which was verified under conditions of temperature and pressure close to the conditions of the hydrogenation reaction. Therefore, a new approach can be used to evaluate and assist in developing new catalysts (Santiago et al.).

Enzymes are suitable candidates for sustainable catalytic process applications (Bonazza et al., 2018; Moreira et al., 2020; Velasco-Lozano et al., 2022). Enzymes have unique catalytic behavior and are widely studied in different catalysis processes (dos Santos et al., 2014; Kurbanoglu et al., 2020; Virgen-Ortíz et al., 2019). Enzymes can act in different types of reactions of industrial interest, such as esterification, transesterification, C-C bond formation, and alcoholysis (Belle and Nijnik, 2014; Prajapati et al., 2022; Liu et al.; González-Davis et al., 2023; Plouhinec et al., 2023; Yamaguchi and Miyazaki, 2023).

Enzymes are used in different industrial processes, which include dairy products (cheese recovery, flavor enhancement, and enzyme-modified cheese (EMC) production), pharmaceuticals (ibuprofen, naproxen), detergents, agricultural products (pesticides, insects), chemicals, oil chemistry (fats and oil hydrolysis and synthesis of biodetergents) (Villalba et al., 2016; Brandão Júnior et al., 2023; Ghattavi and Homaei, 2023; Issaka et al., 2023; Narayanan et al., 2023). Due to their specific properties, in addition to adjusting the reaction conditions of interest, enzymes can be used to replace chemical catalysts. In this context, the modification process of enzymatic functions can happen through alteration of their amino acid residues, side chains, and domain modifications (Biswas et al.). In studies by Biswas et al.), the role of residue modification in the catalytic activity and molecular recognition of an alpha-chymotrypsin (CHT) enzyme in the presence of a formalin covalent crosslinker was performed. The results revealed a reduced catalytic activity after increasing the formalin concentration (Biswas et al.). However, the findings presented in the work of (Biswas et al.) may, in the future, offer information on drug-target interaction, molecular recognition, and macromolecular modification to generate new binding sites for enhanced ligand binding through DNA engineering proteins (Biswas et al.).

Molecular hydrogen (H_2_) is receiving much attention these days as the primary sustainable fuel in different applications in the future. One of the molecular hydrogen production routes involves using precious metal catalysts. The work by Kaim et al. sought to develop alternative non-precious metal catalysts for hydrogen generation, for example, replacing platinum. In the study, the enzyme hydrogenase was used as a model. These studies with manganese catalysts expand the diversity of elements in the periodic table that are favorable to catalyze the hydrogen evolution reaction (Kaim et al.). With this, Kaim et al. opened a new space for further studies on sustainable hydrogen production since the introduction of manganese as an additional metal atom abundant on Earth in the series of mononuclear hydrogen generator catalysts (in addition to Fe, Co., Ni, and Ru). Furthermore, Manganese’s characteristics, including low cost, abundant availability, and a benign environmental profile, make it an exciting candidate for hydrogen (Kaim et al.).

Elucidating the function and catalytic details of enzymes is vital to provide a comprehensive understanding of reaction processes and thereby optimize systems. In order to achieve this goal the studies of Liu et al. (2023b) (Liu et al., 2023b) the state-of-the-art quantum mechanics/molecular mechanics (QM/MM MD) simulation of Born- Oppenheimer was used to systematically understand the mechanism of deAMPylation of AMPylated BiP catalyzed by the enzyme FICD (filamentation induced by cAMP domain protein, also known as HYPE) in detail. The studies were able to show that the transfer of protons from the protonated histidine (His363) in FICD to the AMPylated threonine (Thr518) in BiP initiates the deAMPylation process, instead of the general point of view that refers to a nucleophilic attack of water molecules adding to AMP phosphorus (Liu et al.) (Liu et al., 2023b). Furthermore, it was revealed that the crucial AMPylation inhibitor Glu234, which proved to be essential in the process of bacterial deAMPylation, is possible to alter in mammals (Liu et al.) (Liu et al., 2023b). This research sheds more light on understanding the physiological role of FICD protein and PTMs (posttranslational modifications) (Liu et al.).

This Research Topic covers promising and recent trends in Chemical Reactions and Catalysis for a Sustainable Future. In this opportunity, authors present contributions with original research articles, mini and full reviews, and papers on related Research Topic (Velasco-Lozano et al., 2022). Areas to be covered in the Research Topic include Homogenous catalysts, Heterogenous catalysts, Sustainable alternatives to non-earth great, toxic, and expensive metal catalysts, and catalysis for environmental applications. Essential information is presented here so that researchers can refine their studies in the search for routes with Chemical Reactions and Catalysis for a Sustainable Future. We would like to thank all authors, reviewers, and members of the Editorial Board for their considerable contributions to support the implementation of this special Research Topic.
